# Case Report: Pericardial effusion in late onset neonatal Escherichia coli sepsis

**DOI:** 10.12688/f1000research.167697.1

**Published:** 2025-08-06

**Authors:** N. Missaoui, Rania Ben Rabeh, Azza Hedhili, Salem Yahiaoui, Sofien Atitallah, Olfa Bouyahia, Sonia Mazigh Mrad, Samir Boukthir

**Affiliations:** 1 University of Tunis El Manar Faculty of Medicine of Tunis, Tunis, Tunis, 1007, Tunisia

**Keywords:** pericardial effusion, neonate, E coli, drainage

## Abstract

**Background:**

Pericardial effusion (PE) is a rare condition in neonates and usually due to central venous catheters. Infective pericarditis is an extremely rare condition in neonates.

**Methods:**

We describe a case of a preterm neonate with infective Escherichia coli pericarditis.

**Results:**

A preterm female neonate born at 34 weeks of gestation with a birth weight of 1600 grams was admitted because of respiratory distress. The patient was managed using a high-flow nasal cannula. She did not receive a central venous catheter or antibiotics. The outcome was good and the patient was discharged on day 14. On day 18, she was readmitted because of fever and shortness of breath. Blood sample culture was positive for Escherichia coli. On day 21, the patient presented signs of heart failure. Chest radiography showed cardiomegaly. Cardiac ultrasound showed pre tamponade. Our patient was managed with pericardial drainage and cefotaxime administration. The outcome was good and further follow-up was unremarkable.

**Conclusions:**

Even though rare, infective pericarditis should be suspected in neonates who show deterioration in respiratory and hemodynamic status even if they do not have central venous catheter.

## Introduction

Pericardial effusion (PE) is a rare condition in neonates and usually due to central venous catheters. Symptoms include difficulty to breath and fever. Without appropriate treatment, it can quickly progress to hemodynamic collapse, tamponade, and ultimately, death.

## Case report

A preterm female infant and her twin sister were born at 34 weeks of gestation via an emergency caesarean section due to covid 19 infection in the mother. Serological and prenatal ultrasound findings were normal.

The first twin had severe respiratory distress and was referred to the PICU. The patient was discharged on day 10 of hospitalization. No further symptoms were noted.

Our patient was the second twin . Her birth weight was 1600 g, and Apgar score was 8 at 1 min and 9 at 10 min. She had moderate respiratory distress due to transient tachypnea in the newborn. She was placed on a high-flow nasal cannula for three days. Chest X ray was normal. The patient received fluids through a peripheral venous catheter. The patient did not receive any antibiotics. As her respiration improved, she was placed on room air, fluid infusion was discontinued, and she was gradually fed with milk formula. The patient was discharged on day 14. Four days later (on day 18), she presented to the emergency room with fever and dyspnea. Respiratory rate was 65/minute, she had retractions. Oxygen saturation was 97% in room air. The patient was placed on oxygen via a nasal cannula. Chest X ray was normal. Laboratory tests showed a leukocyte count of 26000/mm ^3^ and an elevated reactive protein C level of 220 mg/l. Initially, a nosocomial infection was suspected, and the patient was received imipenem and amikacin.

**Figure 1.**
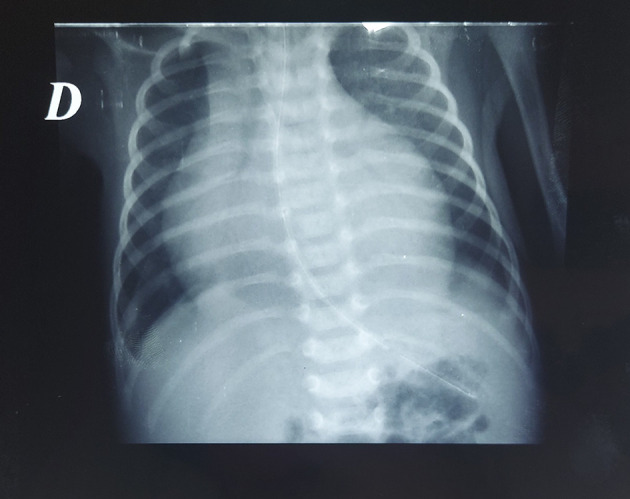
Chest X ray showing cardiomegaly and globular shape of the heart.

**Figure 2.**
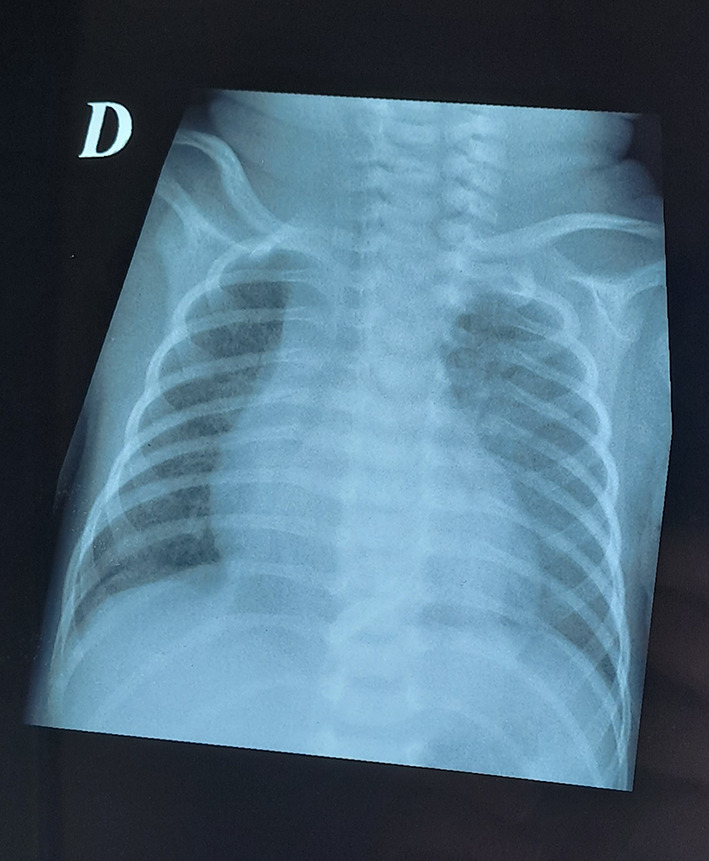
Normal chest X ray after pericardial drainage.

On day 21, two blood cultures obtained on the day of readmission were positive for multidrug-sensitive Escherichia coli (E coli). The patient was then switched to cefotaxime. Two days later (on day 23), our patient developed signs of heart failure with tachycardia, hepatomegaly, tachypnea, and a capillary refill of 4 seconds. Chest radiography revealed significant cardiomegaly with a globular heart shape ( Figure 1). ECG was normal. Cardiac ultrasound revealed pre tamponade. A pericardial drain was inserted surgically, and 8 ml of purulent fluid mixed with blood was aspirated. After pericardial drainage, the respiratory status improved, and the shape of the heart was normal on chest radiography ( Figure 2). No bacteria were detected in pericardial fluid. The pericardial catheter was removed 10 days later. Cefotaxime was continued for a total duration of 14 days. The patient was discharged on day 35 of hospitalization. Further follow-up results were within normal limits.

## Discussion

Pericardial effusion (PE) is rare in neonates. Its clinical presentation depends on the speed and amount of fluid accumulation. ^1^ This can lead to tamponade and even death. The most common cause is iatrogenic because of the central venous catheters. ^2^

In a national American study of all cases of pericardial effusion, the lowest incidence was observed in neonates (0.04%). ^3^ In a literature review, 34 patients were enrolled. The most common causes were central venous catheters (n = 21), Down syndrome (n = 3), and infections (n = 3). One of these patients had a positive blood culture for E. coli. ^2^

Pericardial effusion due to an E. coli infection is rare. To date, only case reports have been published. In 1979, Wynn described purulent pericarditis in a 64 hours aged neonate. Autopsy confirmed the diagnosis of pericardial effusion, and E coli was isolated from the blood culture. ^4^ In 2006, Benjamin described pericarditis in a 10 days old boy. E. coli was isolated from blood samples obtained in the emergency room before referral. He was managed with pericardectomy, pericardial drainage, cefotaxime, and indomethacin. The intraoperative samples were negative.

The management of PE is variable, from surveillance in small asymptomatic effusions to pericardiocentesis, pericardectomy, and pericardial drainage. ^2, 5^ In an American retrospective cohort, pericardial drainage was performed in 8.5% of neonates. ^3^

### Follow-up and outcomes

Outcomes in case reports of infective pericarditis in neonates were good, except in the case of postmortem diagnosis. ^4^ In an American study, the overall mortality was 6.8%, and mortality was the highest among neonates (12.4%). ^3^

Although neonatal pericardial effusion is rare, it should be considered in neonates with cardiac failure, especially in the context of infection. This diagnosis should be considered in neonates who develop signs of deterioration even if they have no central venous catheters. Infective pericarditis in neonates is a rare condition. Management depends on tolerance of the effusion. Mortality was higher in neonates than in the other age groups.

**Use of AI tools:** The authors did not use AI technology in the writing process.

## Ethics and consent

Approval of the local ethics committee of Bechir Hamza Hospital was obtained (n°13/2022). Written informed consent was obtained from the patient’s guardian for the publication of clinical details.

## Data Availability

All data underlying the results are available as part of the article. Figshare: CARE checklist for “Pericardial effusion in late onset neonatal Esherichia coli sepsis”. https://doi.org/10.6084/m9.figshare.29611250.v2 ^6^ All Data are available under the terms of Creative Common Zero (CC0).
