# Case Report: Pericardial effusion in late onset neonatal Escherichia coli sepsis

**DOI:** 10.12688/f1000research.167697.2

**Published:** 2025-09-09

**Authors:** N. Missaoui, Rania Ben Rabeh, Azza Hedhili, Salem Yahiaoui, Sofien Atitallah, Olfa Bouyahia, Sonia Mazigh Mrad, Samir Boukthir

**Affiliations:** 1 University of Tunis El Manar Faculty of Medicine of Tunis, Tunis, Tunis, 1007, Tunisia

**Keywords:** pericardial effusion, neonate, E coli, drainage

## Abstract

**Background:**

Pericardial effusion (PE) is a rare condition in neonates and usually due to central venous catheters. Infective pericarditis is an extremely rare condition in neonates.

**Methods:**

We describe a case of a preterm neonate with *Escherichia coli* pericarditis.

**Results:**

A preterm female neonate born at 34 weeks of gestation with a birth weight of 1600 grams was admitted because of respiratory distress. The patient was managed using a high-flow nasal cannula. She did not receive a central venous catheter or antibiotics. The outcome was good and the patient was discharged on day 14 of life. On day 18, she was readmitted because of fever and shortness of breath. Blood sample culture was positive for *Escherichia coli*. On day 21, the patient presented signs of heart failure. Chest radiography showed cardiomegaly. Cardiac ultrasound showed pre tamponade. Our patient was managed with pericardial drainage and cefotaxime administration. The outcome was good and further follow-up was unremarkable.

**Conclusions:**

Even though rare, infective pericarditis with effusion should be suspected in neonates who show deterioration in respiratory and hemodynamic status even if they do not have central venous catheter.

## Introduction

Pericardial effusion (PE) is a rare condition in neonates. Symptoms include difficulty breathing and fever. Without appropriate treatment, it can quickly progress to hemodynamic collapse, tamponade, and ultimately, death. The most common cause of pericardial effusion in neonates are central venous catheters. It is due to the mechanical and osmotic injury of the thin and immature myocardium in neonates and especially preterm. ^1^

## Case report

A preterm female infant and her twin sister were born at 34 weeks of gestation via an emergency caesarean section due to covid 19 infection in the mother. Maternal serologies (toxoplasmosis, rubella, hepatitis B and syphilis) and prenatal ultrasound findings were unremarkable.

The first twin was small for gestational age and had severe respiratory distress. She was referred to the PICU. She was discharged on day 10 of hospitalization. No further symptoms were noted.

Our patient weighted 1600 g (small for gestational age), and Apgar score was 8 at 1 min and 9 at 10 min. She was referred to out neonatal unit in the pediatric ward for respiratory distress. At presentation, respiratory rate was 62 per minute, heart rate was 138 per minute and oxygen saturation was 97%. Chest radiography was normal. Covid 19 polymerase chain reaction test was negative. She was placed on oxygen therapy via high-flow nasal cannula for three days. The patient received fluids through a peripheral venous catheter. The diagnosis of early onset neonatal infection was discussed but excluded based on: rapid improvement of respiratory status, normal CRP levels and negative blood culture. The patient did not receive any antibiotics. Final diagnosis was transient tachypnea of the newborn. As her respiration improved, she was placed on room air on day 3 of life, fluid infusion was discontinued on day 4 of life, and she was gradually fed with milk formula (breast milk was not available as her family lived from our facility). She was fed initially via gastric tube and gradually via bottle. The patient was discharged on day 14 of life.

On day 18 of life, she presented to the emergency room with fever and dyspnea. Respiratory rate was 65/minute with intercostal recession. Oxygen saturation was 97% in room air. The patient was placed on oxygen via a nasal cannula. Chest radiography was normal. Laboratory tests showed a leukocyte count of 26000/mm ^3^ and an elevated reactive protein C level of 220 mg/l. Initially, a nosocomial infection was suspected, and the patient was received imipenem and amikacin. There was no improvement in her respiratory status.

**Figure 1.**
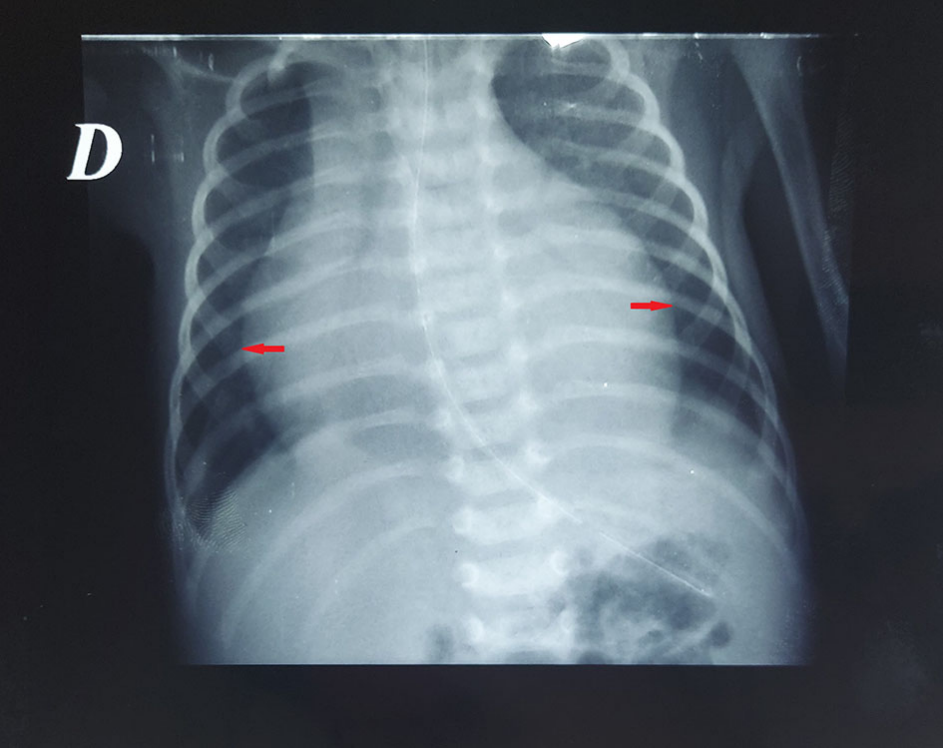
Pre-pericardiocentesis chest radiography demonstrating marked cardiomegaly with globular cardiac silhouette.

On day 21 of life, two blood cultures obtained on the day of readmission were positive for multidrug-sensitive *Escherichia coli* ( *E. coli*). The patient was then switched to cefotaxime. On day 23 of life, our patient developed signs of heart failure with tachycardia, hepatomegaly, tachypnea, and a capillary refill of 4 seconds. Chest radiography revealed cardiomegaly (cardiothoracic ratio 0.67) with a globular heart shape ( Figure 1). Electrocardiogram was normal. Cardiac ultrasound was not available in our hospital so it was performed by cardiologists in another facility. It revealed echogenic, circumferential pericardial effusion (11 mm) and pre tamponade (protodiastolic right ventricle collapse). It also showed a small patent ductus arteriosus. There were no signs of endocarditis.

**Figure 2.**
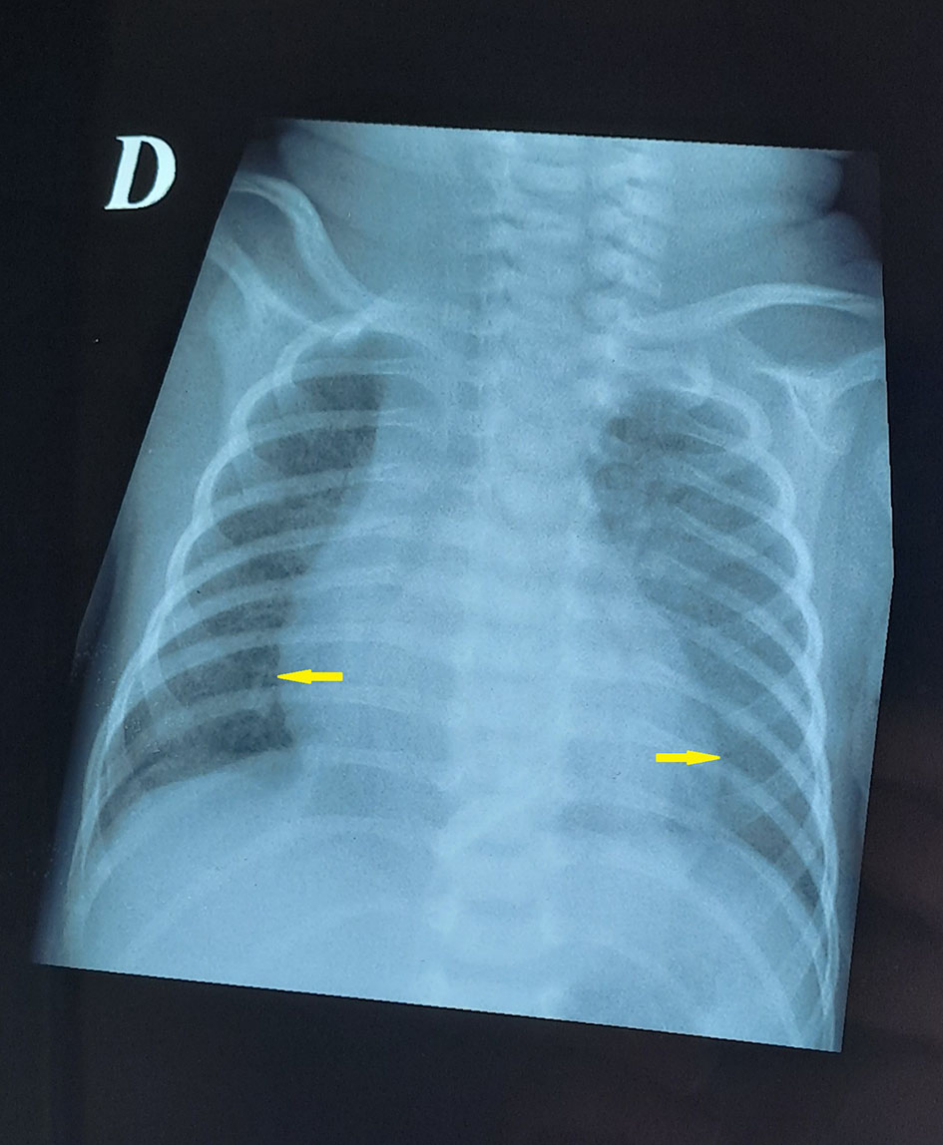
Post-pericardiocentesis chest radiography showing resolution of cardiomegaly with normalization of cardiac silhouette.

A pericardial drain was inserted surgically, and 8 ml of purulent fluid mixed with blood was aspirated. Cytological analysis showed 3 leukocytes per ml. Biochemical analysis was not performed. No bacteria were isolated in pericardial fluid. After pericardial drainage, the respiratory status improved, and the shape of the heart was normal on chest radiography ( Figure 2). The pericardial catheter was removed 10 days later. Cefotaxime was continued for a total duration of 14 days. A second cardiac ultrasound was performed at the age of 32 days. It showed no pericardial effusion. The patient was discharged on day 35 of hospitalization. At discharge, her weight was 2550 g and her physical examination showed normal body temperature, respiratory rate 34 per minute and heart rate 124 per minute. She had regular follow-up until the age of 6 months. At that age, her weight was 6700 g and her physical examination was normal. She was lost of follow-up since.

## Discussion

Pericardial effusion (PE) is rare in neonates. Its clinical presentation depends on the speed and amount of fluid accumulation. ^2^ This can lead to tamponade and even death. The most common cause is iatrogenic because of central venous catheters. ^3^ In a meta-analysis of 21 studies and 99 cases, pooled incidence of PE in neonates who had central venous catheters was 3.8‰. Mortality rate was 2.7%. ^1^

In a national American study of all cases of pericardial effusion, the lowest incidence was observed in neonates (0.04%). ^4^ In a literature review, 34 patients were enrolled. The most common causes were central venous catheters (n = 21), Down syndrome (n = 3), and infections (n = 3). The pathogen agent was isolated in pericardial fluid in only one cas (candida albicans). The second case was caused by *Escherichia coli* isolated in blood culture. In the third case, PE was secondary to parainfluenza virus 3 which was isolated in nasopharyngeal swab. ^3^

Pericardial effusion due to an *E. coli* infection is rare. To date, only case reports have been published. In 1979, Wynn described purulent pericarditis in a 64 hours aged neonate. Autopsy confirmed the diagnosis of pericardial effusion, and *E coli* was isolated from the blood culture. ^5^ In 2006, Benjamin described pericarditis in a 10 days old boy. *E. coli* was isolated from blood samples obtained in the emergency room before referral. He was managed with pericardectomy, pericardial drainage, cefotaxime, and indomethacin. The intraoperative samples were negative. ^6^

Even in *E. coli* sepsis, pericardial effusion remains rare. Lai described the epidemiology of invasive *E. coli* infection in 94 Chinese neonates, including early and late onset disease. No case of pericardial effusion was described. ^7^

In our case, initial suspicion of a nosocomial infection was raised given the patient’s recent hospitalization. The isolation of *E. coli* with multidrug sensitivity underscores the potential for multidrug-resistant strains in neonatal units, especially in settings where broaded-spectrum antibiotics such as carbapenems and aminoglycosides are frequently used empirically. This highlights the importance of strict infection prevention measures, rational antibiotic stewardship and early targeted therapy guided by culture results.

The management of PE is variable, from surveillance in small asymptomatic effusions to pericardiocentesis, pericardectomy, and pericardial drainage. ^3, 8^ In an American retrospective cohort, pericardial drainage was performed in 8.5% of neonates. ^4^

### Follow-up and outcomes

Outcomes in case reports of infective pericarditis in neonates were good, except in the case of postmortem diagnosis. ^5^ In an American study, the overall mortality was 6.8%, and mortality was the highest among neonates (12.4%). ^4^

Although neonatal pericardial effusion is rare, it should be considered in neonates with cardiac failure, especially in the context of infection. This diagnosis should be considered in neonates who develop signs of deterioration even if they have no central venous catheters. Infective pericarditis in neonates is a rare condition. Management depends on tolerance of the effusion. Mortality was higher in neonates than in the other age groups.

### Implication for practice

This case underscores the need for clinicians to maintain high index of suspicion for pericardial effusion in neonates presenting with sepsis and cardiorespiratory deterioration. The absence of central venous catheters cannot reduce the probability. Incorporating point-of-care echocardiography in deteriorating neonates could facilitate earlier detection and intervention. Furthermore, routine review of empirical antibiotic regimens in neonatal sepsis is critical to balance adequate coverage with antimicrobial stewardship.

### Limitations

This report has limitations inherent to single case studies. The follow-up period was relatively short. Ultrasound images were not available in this report.

## Conclusion

Neonatal pericardial effusion due to *Escherichia coli* is an exceedingly rare but potentially life-threatening condition. Early recognition and timely pericardicentesis can be lifesaving.

Recommendations:

• Clinicians should suspect pericardial involvement in neonates with unexplained cardiorespiratory compromise during sepsis, even without central catheters.
• Echocardiography should be integrated in the evaluation of deteriorating neonates with sepsis.
• Infection prevention and control practices should be strengthened in neonatal units to minimize the risk of nosocomial multi-drug resistant infections.
• Empiric antibiotic therapy in neonatal sepsis should be carefully tailored, with early de-escalation based on culture results, to preserve antimicrobial effectiveness.
• Collaborative multicenter registries and pooled case reports are needed to clarify the epidemiology, management and outcomes of neonatal infective pericarditis.

**Use of AI tools:** The authors did not use AI technology in the writing process.

## Ethics and consent

Approval of the local ethics committee of Bechir Hamza Hospital was obtained (n°13/2022). Written informed consent was obtained from the patient’s guardian for the publication of clinical details.

## Data Availability

All data underlying the results are available as part of the article. Figshare: CARE checklist for “Pericardial effusion in late onset neonatal Esherichia coli sepsis”. https://doi.org/10.6084/m9.figshare.29611250.v2 ^9^ All Data are available under the terms of Creative Common Zero (CC0).
