# Supplementary material for: Case Report: Pericardial effusion in late onset neonatal Escherichia coli sepsis
Source: F1000Res. 2025 Sep 9;14:770. Originally published 2025 Aug 6. [Version 2] doi: 10.12688/f1000research.167697.2 (PMC12441665; doi:10.12688/f1000research.167697.2)
Supplement: Peer review history

# Reviewer report: Reviewer response for version 2

**DOI:** 10.5256/f1000research.187677.r412608

**Published:** 2025-09-16

**Authors:** Khadijat Isezuo, Asma'u Adamu

**Affiliations:** 1 Paediatric Cardiology Unit, Department of Paediatrics, Faculty of Clinical Sciences, College of Health Sciences, Usmanu Danfodiyo University, Sokoto, Nigeria; 2 Neonatal Unit, Department of Paediatrics, Faculty of Clinical Sciences, College of Health Sciences, Usmanu Danfodiyo University, Sokoto, Nigeria

Thanks for the opportunity to review this paper after the initial corrections. The authors have adhered to the comments and this has made the report significantly improved. However, there are a few typographical errors and two pending issues pertaining oxygen use with normal saturation and an abnormal Chest X-ray interpretation. They are highlighted in the comments below.

 1.our

 She was referred to **out** neonatal unit in the pediatric ward for respiratory distress. 

 2. nasogastric

 She was fed initially via **gastric** tube

 3. this was not addressed and no reason given

  Oxygen saturation **was 97% i** n room air. The patient was placed **on oxygen** via a nasal cannula.

 4. delete "was"

 Initially, a nosocomial infection was suspected, and the patient **was** received imipenem

 5. lost to

 She was **lost of** follow-up since.

 6. Figure 2 caption

 Figure 2. Post-pericardiocentesis chest radiography showing **resolution of cardiomegaly with normalization of cardiac silhouette.**

 It only shows reduction in cardiomegaly and not a resolution. The cardiac silhouette is still not completely normal. So caption as a reduction. Moreover, the cardiothoracic ratio was not measured to compare both.

 7. case

 The pathogen agent was isolated in pericardial fluid in only one **cas** (candida....

 8. pericardiectomy

 He was managed with **pericardectomy,**

 9. broad-spectrum

 The isolation of *E. coli* with multidrug sensitivity underscores the potential for multidrug-resistant strains in neonatal units, especially in settings where **broaded-spectrum** antibiotics such as carbapenems and aminoglycosides are frequently used empirically.

 10. Cardiac ultrasound

**Ultrasound** images were not available in this report.

Are enough details provided of any physical examination and diagnostic tests, treatment given and outcomes?

Partly

Is the case presented with sufficient detail to be useful for other practitioners?

Partly

Is sufficient discussion included of the importance of the findings and their relevance to future understanding of disease processes, diagnosis or treatment?

Partly

Is the background of the case’s history and progression described in sufficient detail?

Yes

Reviewer Expertise:

Paediatric cardiology, Neonatology, Infectious disease, Nutrition

We confirm that we have read this submission and believe that we have an appropriate level of expertise to confirm that it is of an acceptable scientific standard.

# Reviewer report: Reviewer response for version 2

**DOI:** 10.5256/f1000research.187677.r412607

**Published:** 2025-09-16

**Authors:** Lamin Makalo

**Affiliations:** 1 University of the Gambia, Serrekunda, Banjul, The Gambia

Good enough to be indexed,

 Dr Lamin Makalo

 University of The Gambia.

Are enough details provided of any physical examination and diagnostic tests, treatment given and outcomes?

Partly

Is the case presented with sufficient detail to be useful for other practitioners?

Yes

Is sufficient discussion included of the importance of the findings and their relevance to future understanding of disease processes, diagnosis or treatment?

Partly

Is the background of the case’s history and progression described in sufficient detail?

Yes

Reviewer Expertise:

Paediatric Hematology, Pulmonology, Cardiology, Nutrition and Gastroenterology

I confirm that I have read this submission and believe that I have an appropriate level of expertise to confirm that it is of an acceptable scientific standard.

# Reviewer report: Reviewer response for version 1

**DOI:** 10.5256/f1000research.184830.r406263

**Published:** 2025-09-01

**Authors:** Khadijat Isezuo, Asma'u Adamu

**Affiliations:** 1 Paediatric Cardiology Unit, Department of Paediatrics, Faculty of Clinical Sciences, College of Health Sciences, Usmanu Danfodiyo University, Sokoto, Nigeria; 2 Neonatal Unit, Department of Paediatrics, Faculty of Clinical Sciences, College of Health Sciences, Usmanu Danfodiyo University, Sokoto, Nigeria

Dr Khadijat O. Isezuo **(Reviewer A)**

**General comments: **

1. This is an interesting case report which clearly depicts the evolution of symptomatology in a preterm neonate eventually leading to a diagnosis of pericardial effusion. A positive blood culture yielded E.coli and was attributed to be the cause of pericarditis though the pericardial fluid was sterile despite being purulent. It brings to bear that the relatively immunodeficient neonate can manifest deep seated foci of sepsis and unstable cardiorespiratory symptomatology should be thoroughly investigated.
2. However, the possibility of infective endocarditis was not  mentioned as a possible differential and excluded with the Dukes criteria.[1]
3.  The overall status and outcome of the other twin could also be mentioned.
4. The COVID status of the babies should be excluded too

 

1. **Title **
  1. It is ok except the possibility of early onset sepsis in the child due to timing of initial symptoms and lack of antibiotics

 

1. **Abstract: **

1. Introduction _line 1: Adjust to “Usually due to complication of Central Venous Catheter”
2. ‘Remove infective’ from methods
3. Conclusion: infective pericarditis with effusion

 

1. **Introduction **
  1. it is too short and has no references
  2. explain briefly how Central venous catheter causes pericardial effusion
  3. one can mention other causes of neonatal pericardial effusion ie frequent causes here to expatiate on how common or rare infective causes are.

 

1. **Case Report**
  1. The serological findings--- Do they refer to the mother or babies? Which test specifically were done? Any possibility of covid infection contributing to respiratory distress in them? This can be explored in the discussion if word count allows for it. [2,3,4]
  2. What was the 1 ^st^ twin diagnosis and anthropometry and were they AGA or SGA or LGA?
  3. Where was this patient managed if the twin was in PICU? What were her own investigations if related?
  4. The 3 ^rd^ paragraph of the case report is too choked up. It contains the events at birth, those during the 1 ^st^ admission till discharge, those on readmission. It makes it a bit difficult to follow. It is better to separate them, possibly sub-headers if guidelines allow.
  5. During the 1 ^st^ admission, diagnosis was TTN, this seems odd for that level of prematurity. Was sepsis considered then? Specifically, early onset sepsis? Was there any other risk in the mother? Any sepsis screen then? Despite the length of stay, why no antibiotics?
  6. At what day was the infusion discontinued?
  7. A table showing the summary of investigation results and reference changes would be useful rather than being subsumed within the text
  8. She had 4 chest x-rays in how many days? Safety? Present the normal x-rays images chronologically side by side before the abnormal one. In the text, mention as follows—repeat chest x-ray on day*** of life so as to give more context. Chest x-ray with pericardial effusion also has minimal pleural effusion on the right side. What were the cardiothoracic ratios?
  9. Is there any ECG result? Why echo not done earlier and no echo image is provided at diagnosis and at resolution? The echo dimensions of the free space occupied by fluid can also be mentioned. There is no specific mention if intra-cardiac defect was excluded eg a PDA at this age in a preterm. Likewise as mentioned in the general comment, infective endocarditis (gram negative) was not fully explored and excluded with the criteria. [1]
  10. What were the parameters at discharge eg weight, respiratory rate, heart rate, temperature?

 

1. **Discussion**
  1. As acute pericarditis is a major clinical manifestation of long COVID-19 syndrome, was there any link to their mother’s status? [5]
  2. Discussion did not on dwell on neonatal causes of pericardial effusion.
  3. In the 3rd paragraph, no reference number for Benjamin’s findings.
  4. Find out if this very recent study includes neonatal cases and reference it --------[6] Richard, B., Preka, E., Grapin, M. Et al. Pericardial effusion in pediatric Shiga toxin-producing E. Coli hemolytic uremic syndrome: a French multicentre study. Pediatr Nephrol (2025). https://doi.org/10.1007/s00467-025-06877-0
  5. The references are too few. This can be used to support or disprove the rarity. Kindly check it.----[7] Lai, J., Zhu, Y., Tang, L. et al. Epidemiology and antimicrobial susceptibility of invasive Escherichia coli infection in neonates from 2012 to 2019 in Xiamen, China. BMC Infect Dis 21, 295 (2021). https://doi.org/10.1186/s12879-021-05981-4

 

1. **Others**
  1. What of confidentiality in ethics statement? And other ethical issues.

**Final recommendation:**

 To accept after the above revisions are fairly addressed.

 Prof Asma’u Adamu **(Reviewer B)**

1. **Title**

1. The title can be modified to “Purulent pericardial effusion in a preterm neonate with late onset Escherichia coli septicaemia”

 

1. **Abstract**

1. That was divided into two subsections.
2. It is not necessary - to remove background, methods and result and allow the sentences to flow

 

1. **Keywords**

1. Delete drainage

 

1. **Case Report**

1. The first twin that had severe respiratory distress, what was the diagnosis, birth, PCV and APGAR?
2. Correct to transient Tachypnoea of the new born not transient tachypnoea in the newborn.
3. Delete moderate respiratory distress – leave as respiratory distress
4. She was placed in a high-flow nasal cannula oxygen therapy – add oxygen therapy; do so also in the abstract
5. The patient receive fluid through a peripheral venous catheter – correct to the patient received fluid via a peripheral venous line
6. Add the oxygen saturation of the patient in a room air at presentation before commencement of oxygen and heart rate
7. Why feeding with milk formula not breast milk; add reason for not giving breastmilk.
8. Four days later (on day 18) add (on day 18 of life)
9. Add the specific chest retractions observed in this patient like intercostal recession, subcostal recession and so on.
10. Delete that she had retraction
11. The patient was placed on oxygen with a normal oxygen saturation of 97%. Delete the patient was placed on oxygen
12. And the patient was received Imipenem and Amikacin – correct to the patient received Imipenem and Amikacin
13. On day 21, the two blood cultures that were obtained, add their antibiotic sensitivity pattern
14. The cefotaxime that was switched to, is it based on the antibiotic sensitivity?
15. Add (23 days of life)
16. The hepatomegaly, is it tender? If tender add
17. Chest radiography revealed significant cardiomegaly – delete significant
18. No bacteria was detected- replace detected with isolated

 

1.  
**Discussion**

1. To date only case reports
2. Are they the only case reports on similar case? If yes add to date the only case reports
3. You have compared your findings with other reports, what made your case different, if any add to the discussion
4. Highlight diagnostic delay, patient developed tamponade on day 23 despite antibiotic could an earlier, ECHO have helped
5. Also discuss lessons for clinician. In neonate with sepsis and cardiac signs, early ECHO is crucial in the absence of central lines
6. Add also in the discussion the reason for negative culture of the pericardial fluid such as prior use of antibiotics, inflammation and so on. Could a pericardial biopsy and PCR have helped? [8]

 

1. **References**

1. Some of the references were too old, use recent references

**Final recommendation:**

 To accept after revisions

Are enough details provided of any physical examination and diagnostic tests, treatment given and outcomes?

Partly

Is the case presented with sufficient detail to be useful for other practitioners?

Partly

Is sufficient discussion included of the importance of the findings and their relevance to future understanding of disease processes, diagnosis or treatment?

Partly

Is the background of the case’s history and progression described in sufficient detail?

Yes

Reviewer Expertise:

Paediatric cardiology, Neonatology, Infectious disease, Nutrition

We confirm that we have read this submission and believe that we have an appropriate level of expertise to confirm that it is of an acceptable scientific standard, however we have significant reservations, as outlined above.

## References

[1] : Infective Endocarditis, a Rare Complication of Late Neonatal Group B Strep Sepsis. *Frontiers in Pediatrics* .2018;6: 10.3389/fped.2018.00274 10.3389/fped.2018.00274 30338251 PMC6178889

[2] : Maternal COVID-19 infection and risk of respiratory distress syndrome among newborns: a systematic review and meta-analysis. *BMC Infectious Diseases* .2024;24(1) : 10.1186/s12879-024-10161-1 10.1186/s12879-024-10161-1 39563236 PMC11577808

[3] : Impacts of maternal COVID-19 during pregnancy on neonatal health and epidemiology. *Clinical and Experimental Pediatrics* .2024;67(3) : 10.3345/cep.2023.01452 149-151 10.3345/cep.2023.01452 PMC1091545738186261

[4] : Association between maternal coronavirus disease 2019 and transient tachypnea of the newborn: a single-center study. *Clinical and Experimental Pediatrics* .2023;66(11) : 10.3345/cep.2023.00563 493-500 10.3345/cep.2023.00563 PMC1062602337873564

[5] : 109: CARDIAC TAMPONADE: A DELAYED COMPLICATION OF COVID-19. *Critical Care Medicine* .2022;50(1) : 10.1097/01.ccm.0000806760.98752.f9 10.1097/01.ccm.0000806760.98752.f9

[6] : Pericardial effusion in pediatric Shiga toxin-producing E. coli hemolytic uremic syndrome: a French multicentre study. *Pediatric Nephrology* .2025; 10.1007/s00467-025-06877-0 10.1007/s00467-025-06877-0 40694098

[7] : Epidemiology and antimicrobial susceptibility of invasive Escherichia coli infection in neonates from 2012 to 2019 in Xiamen, China. *BMC Infectious Diseases* .2021;21(1) : 10.1186/s12879-021-05981-4 10.1186/s12879-021-05981-4 33757434 PMC7988952

[8] : Culture‐negative group B streptococcal pericarditis: A case report and literature review of the diagnostic use of polymerase chain reaction. *Clinical Case Reports* .2019;7(3) : 10.1002/ccr3.2017 509-514 10.1002/ccr3.2017 PMC640615530899483

# Response

**Published:** 2025-09-03

**Authors:** Nada Missaoui

**Affiliations:** University of Tunis El Manar Faculty of Medicine of Tunis, Tunis, Tunis, Tunisia

Dear Dr Isezuo,

 We sincerely thank your for evaluating our manuscript.

 Response to comment 1 (Introduction): More details were added as well as the role of central venous catheters.

 Response to comment on Main body: more information about maternal serologies, twin anthropometry were added. The diagnosis of early onset neonatal infection (during the first hospitalization) was discussed and excluded. Infusion was discontinued on day 4. Details have been added to the manuscript.

 Our patient had 4 X rays during her two hospitalizations (45 days). We believe that her safety was not compromised.

 ECG was normal (already mentioned in the original version). Cardiac ultrasound is not available in our facility. All the findings of cardiac ultrasound were added to the manuscript (this detail as well as was added to the manuscript).

 Physical examination parameters were mentioned in the revised version of the manuscript.

 We appreciate your suggestions regarding discussion and recent articles. They enhanced the quality of our article.

# Reviewer report: Reviewer response for version 1

**DOI:** 10.5256/f1000research.184830.r406267

**Published:** 2025-08-21

**Authors:** Lamin Makalo

**Affiliations:** 1 University of the Gambia, Serrekunda, Banjul, The Gambia

I’ve reviewed the case report titled **“Pericardial effusion in late onset neonatal Escherichia coli sepsis” (F1000Research, 2025)**.

 Here’s my assessment of its fitness for publication:

**Strengths**

1. **Relevance and Novelty**
  - Infective pericarditis due to *E. coli* in neonates is very rare; documenting such a case adds value to neonatal infectious disease literature.
  - The case highlights the importance of suspecting pericardial effusion even without a central venous catheter.
2. **Structure and Clarity**
  - Follows the expected **case report format**: Abstract, Introduction, Case description, Discussion, Conclusion, Ethics, and Data availability.
  - Clinical timeline is clear: initial presentation, discharge, readmission, infection, diagnosis of pericardial effusion, management, and outcome.
  - Ethical approval and informed consent are explicitly stated.
3. **Use of Literature**
  - References are relevant and include both classic and recent reports.
  - Situates the case in the context of previously reported neonatal pericardial effusions.
4. **Compliance**
  - CARE guidelines checklist has been attached (per Fig share reference).
  - Transparency regarding AI use (declared none).
  - No conflicts of interest or funding bias.

**Weaknesses / Areas for Improvement**

1. **Language and Grammar**
  - Several sentences are grammatically awkward or lack fluency (e.g., “difficulty to breath” should be “difficulty breathing”).
  - Some inconsistencies in spacing and punctuation.
2. **Case Details**
  - **Pericardial fluid analysis**: It was stated no bacteria were detected, but biochemical/cytological analysis details (protein, glucose, cell count) are missing. These would strengthen diagnostic certainty.
  - **Antibiotic regimen**: The switch from imipenem/amikacin to cefotaxime is described, but no rationale is given for narrowing therapy despite *E. coli* being multidrug-sensitive.
  - **Twin context**: The first twin’s outcome is described but seems peripheral—clarify its relevance.
  - **Follow-up duration**: States “further follow-up was unremarkable” but doesn’t indicate for how long (weeks/months). This limits assessment of long-term prognosis.
3. **Figures**
  - Only X-rays (before and after drainage) are provided. Cardiac ultrasound images would have added diagnostic weight.
4. **Discussion**
  - Could be expanded to discuss:
    - Pathophysiology of *E. coli* spread to the pericardium.
    - Why neonates may be more vulnerable.
    - Broader implications for neonatal sepsis management in resource-limited settings.
5. **Minor Issues**
  - Typo in title and reporting guideline reference: “Esherichia coli” instead of **Escherichia coli**.
  - Some references (e.g., Ferraz Liz et al.) could benefit from more context when cited.

**Conclusions**

 Even though rare, infective pericarditis should be suspected in neonates who show deterioration in respiratory and hemodynamic status even if they do not have a central venous catheter. This case also highlights concerns for multidrug resistance in neonatal sepsis and the importance of antibiotic stewardship.

  I recommend the following for the figures

**Figure 1.** Pre-pericardiocentesis chest radiograph demonstrating marked cardiomegaly with a globular cardiac silhouette (arrows indicate the enlarged cardiac borders).

**Figure 2.** Post-pericardiocentesis chest radiograph showing resolution of cardiomegaly with normalization of cardiac silhouette (arrows indicate normalized heart borders).

**Discussion (MDR concern & implications)**

 I suggest adding after the paragraph describing *E. coli* reports:

*“In our case, initial suspicion of a nosocomial infection was raised given the patient’s recent hospitalization. The isolation of E. coli with multidrug sensitivity underscores the potential for multidrug-resistant (MDR) strains in neonatal units, especially in settings where broad-spectrum antibiotics such as carbapenems and aminoglycosides are frequently used empirically. Although our isolate was susceptible, vigilance is warranted as MDR E. coli pericarditis could severely limit therapeutic options. This highlights the importance of strict infection prevention measures, rational antibiotic stewardship, and early targeted therapy guided by culture results.”*

**Implications for practice**

 I suggest inserting in the  Discussion/Conclusion transition:

*“This case underscores the need for clinicians to maintain a high index of suspicion for pericardial effusion in neonates presenting with sepsis and cardiorespiratory deterioration, even in the absence of central venous catheters. The overlap between neonatal sepsis and cardiac failure may delay recognition of pericardial involvement. Incorporating point-of-care echocardiography in deteriorating neonates could facilitate earlier detection and intervention. Furthermore, routine review of empirical antibiotic regimens in neonatal sepsis is critical to balance adequate coverage with antimicrobial stewardship.”*

**Limitations**

 I suggest adding this before conclusion:

 “This report has limitations inherent to single case studies. Pericardial fluid biochemical and cytological analyses were not performed, limiting full characterization of the effusion. The follow-up period was relatively short, and longer-term outcomes could not be assessed. Ultrasound images were not archived for inclusion in this report, which would have provided stronger diagnostic demonstration.”

**Conclusion and Recommendations**

 The following is suggested to strengthen the conclusion and therefore give recommendations to strengthen the report

**Conclusion**

 Neonatal pericardial effusion due to *Escherichia coli* is an exceedingly rare but potentially life-threatening condition. Early recognition and timely pericardiocentesis can be lifesaving.

**Recommendations**

- Clinicians should suspect pericardial involvement in neonates with unexplained cardiorespiratory compromise during sepsis, even without central catheters.
- Echocardiography should be integrated into the evaluation of deteriorating neonates with sepsis.
- Infection prevention and control practices should be strengthened in neonatal intensive care settings to minimize the risk of nosocomial MDR infections.
- Empiric antibiotic therapy in neonatal sepsis should be carefully tailored, with early de-escalation based on culture results, to preserve antimicrobial effectiveness.
- Collaborative multicenter registries and pooled case reports are needed to clarify the epidemiology, management, and outcomes of neonatal infective pericarditis.

Are enough details provided of any physical examination and diagnostic tests, treatment given and outcomes?

Partly

Is the case presented with sufficient detail to be useful for other practitioners?

Yes

Is sufficient discussion included of the importance of the findings and their relevance to future understanding of disease processes, diagnosis or treatment?

Partly

Is the background of the case’s history and progression described in sufficient detail?

Yes

Reviewer Expertise:

Paediatric Hematology, Pulmonology, Cardiology, Nutrition and Gastroenterology

I confirm that I have read this submission and believe that I have an appropriate level of expertise to confirm that it is of an acceptable scientific standard, however I have significant reservations, as outlined above.

# Response

**Published:** 2025-09-03

**Authors:** Nada Missaoui

**Affiliations:** University of Tunis El Manar Faculty of Medicine of Tunis, Tunis, Tunis, Tunisia

Dear Dr Makalo,

 We sincerely appreciate your constructive feedback. Your insightful comments have helped us refine and enhance the quality of our manuscript.

 Response to comment 1: We have made corrections to grammar and punctuations mistakes.

 Response to comment 2: Details of pericardial fluid analysis, the rational of antibiotic switch and follow-up have been added to the manuscript.

 Response to comment 3: Cardiac ultrasound was not performed in our facility (this detail was added to the manuscript). Unfortunately, no images were provided from cardiologists.

 Response to comments on discussion: we appreciate your suggestions. We have included them in the manuscript.
